# Supplementary material for: The lower COVID-19 related mortality and incidence rates in Eastern European countries are associated with delayed start of community circulation
Source: PLoS One. 2020 Dec 3;15(12):e0243411. doi: 10.1371/journal.pone.0243411 (PMC7714339; doi:10.1371/journal.pone.0243411)
Supplement: S1 Data — (DOC) [file pone.0243411.s001.doc]

**Annex.** Dataset used for the analyses

| Countries | Date of first confirmed case | Date when first 100 cases recorded | Days from Jan 22^st^ to date 100 first cases recorded | Life expectancy | Incidence  /million | Mortality  /million | Outpatients contacts per person per year |
| --- | --- | --- | --- | --- | --- | --- | --- |
| Italy | 29-Jan | 22-Feb | 31 | 83 | 3523 | 485 | 6.8 |
| France | 23-Jan | 29-Feb | 38 | 83 | 2613 | 391 | 6.3 |
| Germany | 26-Jan | 01-Mar | 39 | 81 | 1998 | 83 | 9.9 |
| Spain | 30-Jan | 01-Mar | 39 | 83 | 5426 | 553 | 7.6 |
| UK | 31-Jan | 04-Mar | 42 | 81 | 2872 | 433 | 5.0 |
| Switzerland | 25-Feb | 04-Mar | 42 | 84 | 3473 | 207 | 4.0 |
| Sweden | 31-Jan | 05-Mar | 43 | 83 | 2368 | 291 | 2.9 |
| Norway | 26-Feb | 05-Mar | 43 | 83 | 1467 | 40 | 4.3 |
| Netherland | 26-Feb | 05-Mar | 43 | 82 | 2411 | 304 | 8.0 |
| Belgium | 04-Feb | 06-Mar | 44 | 82 | 4382 | 720 | 7.4 |
| Austria | 25-Feb | 08-Mar | 46 | 82 | 1741 | 68 | 6.8 |
| Denmark | 27-Feb | 09-Mar | 47 | 81 | 1716 | 87 | 4.4 |
| Israel | 21-Feb | 11-Mar | 50 | 83 | 1885 | 27 | 6.2 |
| Greece | 26-Feb | 11-Mar | 50 | 81 | 255 | 14 | 4.0 |
| Iceland | 28-Feb | 11-Mar | 50 | 83 | 5272 | 29 | 5.9 |
| Czechia | 01-Mar | 11-Mar | 50 | 79 | 741 | 24 | 11.1 |
| Finland | 29-Jan | 12-Mar | 51 | 82 | 1006 | 45 | 4.2 |
| Portugal | 02-Mar | 12-Mar | 51 | 82 | 2568 | 107 | 4.1 |
| Slovenia | 04-Mar | 12-Mar | 51 | 81 | 697 | 48 | 6.6 |
| Romania | 26-Feb | 13-Mar | 52 | 75 | 733 | 45 | 4.8 |
| Irland | 29-Feb | 13-Mar | 52 | 83 | 4452 | 271 | 5.7 |
| Estonia | 27-Feb | 14-Mar | 53 | 79 | 1291 | 41 | 6.3 |
| Poland | 04-Mar | 14-Mar | 53 | 78 | 389 | 19 | 7.2 |
| Luxemburg | 29-Feb | 16-Mar | 55 | 82 | 6134 | 153 | 5.9 |
| Russia | 30-Jan | 16-Mar | 55 | 73 | 1137 | 11 | 8.4 |
| Slovakia | 06-Mar | 17-Mar | 56 | 77 | 262 | 5 | 11.3 |
| Turkey | 09-Mar | 17-Mar | 56 | 77 | 1535 | 42 | 8.3 |
| Armenia | 01-Mar | 18-Mar | 57 | 75 | 939 | 13 | 4.1 |
| Serbia | 06-Mar | 18-Mar | 57 | 76 | 1121 | 23 | 7.8 |
| Croatia | 25-Feb | 19-Mar | 58 | 78 | 516 | 21 | 6.3 |
| Bulgaria | 06-Mar | 19-Mar | 58 | 75 | 256 | 12 | 5.9 |
| Latvia | 02-Mar | 20-Mar | 59 | 75 | 477 | 9 | 5.9 |
| N. Macedonia | 26-Feb | 21-Mar | 60 | 76 | 739 | 42 | 7.0 |
| Lithuania | 28-Feb | 21-Mar | 60 | 76 | 525 | 18 | 8.7 |
| Hungary | 04-Mar | 21-Mar | 60 | 76 | 322 | 39 | 11.8 |
| Bosnia Herzegovina | 05-Mar | 21-Mar | 60 | 77 | 606 | 26 | 5.2 |
| Moldova | 07-Mar | 22-Mar | 61 | 72 | 1082 | 34 | 6.4 |
| Cyprus | 09-Mar | 22-Mar | 61 | 81 | 731 | 12 | 2.2 |
| Malta | 07-Mar | 23-Mar | 62 | 82 | 1096 | 11 |  |
| Albania | 08-Mar | 23-Mar | 62 | 78 | 289 | 11 | 2.5 |
| Ukraine | 04-Mar | 24-Mar | 63 | 72 | 301 | 7 | 9.3 |
| Azerbaijan | 28-Feb | 25-Mar | 64 | 73 | 210 | 3 | 4.7 |
| Kazakhstan | 13-Mar | 26-Mar | 65 | 73 | 231 | 2 | 4.6 |
| Kosovo | 13-Mar | 27-Mar | 66 | 72 | 464 | 14 |  |
| Uzbekistan | 15-Mar | 27-Mar | 66 | 72 | 67 | 0.3 | 8.8 |
| Belarus | 28-Feb | 29-Mar | 68 | 74 | 2038 | 12 | 13.5 |
| Georgia | 26-Feb | 30-Mar | 69 | 74 | 153 | 2 | 3.3 |
| Montenegro | 16-Mar | 30-Mar | 69 | 77 | 516 | 13 | 7.1 |
| Kirgizstan | 18-Mar | 30-Mar | 69 | 71 | 134 | 2 | 2.5 |
| Tajikistan | 30-Apr | 03-May | 73 | 71 | 40 | 0.8 | 4.3 |
